# The prevalence of anxiety and depression in people with age-related macular degeneration: a systematic review of observational study data

**DOI:** 10.1186/1471-2415-14-78

**Published:** 2014-06-12

**Authors:** Sarah R Dawson, Christian D Mallen, Matthew B Gouldstone, Robert Yarham, Gemma Mansell

**Affiliations:** 1 Arthritis Research UK Primary Care Centre, Primary Care Sciences, Keele University, Keele, Staffordshire ST5 5BG, UK

**Keywords:** Macular degeneration, Depression, Anxiety, Prevalence, Epidemiology

## Abstract

**Background:**

Comorbid mental health problems have been shown to have an adverse effect on the quality of life of people with common eye disorders. This study aims to assess whether symptoms of anxiety and/or depression are more prevalent in people with age-related macular degeneration (AMD) than in people without this condition.

**Methods:**

A systematic search of electronic databases (Medline, CINAHL, EMBASE, PsycINFO) from inception to February 2012 was conducted to identify studies of AMD populations which measured symptoms of anxiety and/or depression. Reference checking of relevant articles was also performed. Data on the study setting, prevalence and how anxiety and depression were measured were extracted from the papers. Critical appraisal was performed using the Critical Appraisal Skills Programme (CASP) tools.

**Results:**

A total of 16 papers were included in the review, from an original search result of 597. The prevalence estimates, taken from nine cross-sectional and cohort studies, ranged from 15.7%-44% for depressive symptoms and 9.6%-30.1% for anxiety symptoms in people with AMD. The seven case–control studies found that people with AMD were more likely to experience symptoms of depression compared with those without AMD, but not more likely to experience symptoms of anxiety.

**Conclusions:**

Overall, the evidence suggests that symptoms of depression are more prevalent amongst AMD populations than anxiety symptoms. The heterogeneity of the studies included in this review means that it is difficult to draw strong conclusions as to the true estimates of depression and anxiety symptoms in AMD populations and prevented formal meta-analysis. Further research which specifies clinical anxiety and gives clear definitions as to the type of AMD being investigated is required.

## Background

Age-related macular degeneration (AMD) is a condition affecting between 20 to 25 million people worldwide and is the most common form of visual impairment in people aged over 50 in the United Kingdom [1,2]. Symptoms of AMD, including difficulty in reading text, recognising faces and completing general house work [3], may directly lead to increased disability which could in turn lead to an increase in symptoms of depression and anxiety [4]. Depression and anxiety could be also be exacerbated by the social isolation caused by AMD or by the need to give up work [5]. Ranibizumab is currently the only on license drug available in the UK to treat neovascular AMD [6,7], and an association of poor vision and increased mental health issues may be an additional factor that could be used when justifying this high cost treatment.

Studies of clinical mood disorders have prevalence estimates of between 8-12% in the general UK population [8] and up to 13% [9] in UK primary care populations. The number of people with non-clinical mood symptoms is likely to be much higher. The prevalence of psychiatric symptoms is likely to be different in community compared to clinical settings, with prevalence rates generally increasing through primary, secondary and tertiary care. Comorbid mental illness is known to have a significant adverse effect on quality of life in a number of physical conditions [10-12], including AMD [3,13-17], meaning that its detection and management represents a priority for clinicians and patients alike. A previous systematic review [16] of quality of life in people with AMD provides a brief summary of the evidence for anxiety and depressive symptoms but the present review updates this evidence and reviews it in more detail.

The aim of this study is to assess whether symptoms of anxiety and/or depression are more prevalent in people with AMD than in those without.

## Methods

The PRISMA guidelines [18] were followed for this review and the sections below are set out according to these guidelines.

### Eligibility criteria

Papers which reported the prevalence of anxiety, depression or both in adults aged 18 years and above with AMD (cross-sectional or cohort studies), or reported anxiety and/or depression scores of both AMD sufferers and a comparison group (case–control studies), were included. Any setting was permitted (primary care, secondary care or general population), although the search was limited to English language papers. Clinical trials were excluded as they include highly selected populations.

### Information sources

The following databases were searched from inception to February 2012: CINAHL, EMBASE, PsycINFO and Medline. The reference lists of relevant papers were also searched for further papers.

### Search

The search was developed by a group with expertise in clinical medicine, psychology and systematic reviewing and included terms for anxiety, depression and AMD.

Search strategy for electronic databases

“worry” OR “panic” OR “anxiety” OR “Nervousness” OR “concern” OR “apprehension” OR “angst” OR “fear” OR “disquiet” OR “fretfulness” OR “unease” OR “despair” OR “sadness” OR “misery” OR “hopelessness” OR “melancholy” OR “dejection” OR “depression” OR “unhappiness”

AND

“macular” OR “degeneration” OR “AMD” OR “ARMD”.

### Study selection

After title scanning, abstracts from the electronic databases thought to be relevant were retrieved for closer inspection. If deemed to meet the inclusion criteria, the full text of the article was located. The reference lists of all full text papers from the electronic search were scanned for further relevant articles, which were processed in the same way as those retrieved from the electronic search.

### Data collection process

Information from all papers found to meet the inclusion criteria was entered into a data extraction table created using Microsoft Word. Details included the study setting, the prevalence and the method of assessment of anxiety and/or depression. Data was independently extracted by two reviewers to ensure accuracy.

### Risk of bias in individual studies

All included studies were subjected to methodological critical appraisal using the Critical Appraisal Skills Programme (CASP). These tools contain a series of questions to assess how effectively the participants in each of the studies were selected, the main results of the study, the reliability of these results and whether the results can be used to benefit the local population. CASP was chosen as it is a comprehensive and detailed appraisal tool suitable for assessing the studies included in the review. Different versions of the tool are tailored to different study designs. A score is generated by summing the number of positive responses to each question, with a higher number indicating higher quality. Each article was assessed by three independent reviewers (SRD, MBG, RY) and any differences in score were resolved by discussion with a 4^th^ reviewer (CDM). The total CASP score each paper received is included in the data extraction table (Additional file 1).

### Summary measures

The prevalence of anxiety and/or depression was extracted from the cross-sectional and cohort studies, and the *p*-value indicating whether anxiety and/or depression were statistically significantly higher in the AMD population compared to the control group was taken from the case–control studies.

### Risk of bias across studies

**Figure 1:**
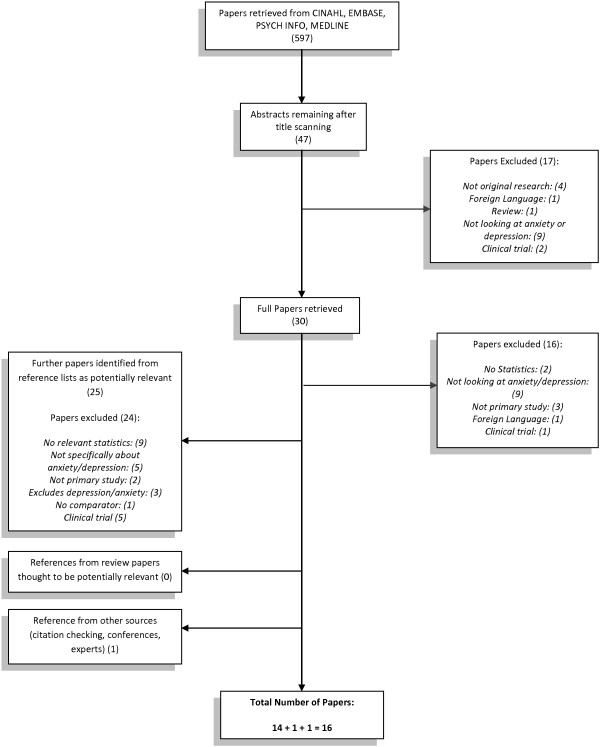
Flowchart for how articles were chosen for inclusion.

Non-English language papers could not be included due to lack of translation resources, but the number of papers excluded for this reason was included in the study flowchart (Figure 1) so the potential impact of this could be examined. It is also possible that studies that did not find a high prevalence of anxiety or depression are less likely to be published than those that did find a high prevalence.

## Results

### Study selection

Of the 597 electronic search results, 30 full papers were retrieved. The reference lists of each of these papers were scanned and a further 25 papers were identified as possibly relevant. Of the original 30 studies, 14 were relevant and one of the 25 later identified was relevant. A final paper was identified from an expert in the literature, giving a total of 16 papers (see Figure 1).

### Study characteristics

The detailed results of the review can be found in Additional file 1. Out of the 16 studies included (including between 51 and 65,404 participants), nine were conducted in the United States, one in Canada, one in Australia and one was a multi-country study involving France, Germany and Italy. Four of the remaining studies were part of a different multi-country study; one of these papers contained the overall results for Canada, France, Germany, Spain and the UK [19] and the individual results from the UK [20], Spain [21] and Canada [3] make up the remaining three papers. Three of the papers were cohort studies [13,14,22], seven were case–control studies [3,19-21,23-25] and the remaining six were cross-sectional surveys [15,26-30].

### Measures of anxiety and depression

Anxiety and depression were measured using a number of different tools, the most popular being the Hospital Anxiety and Depression Scale (HADS) [3,19-21,28] and the Centre for Epidemiological Studies- Depression scale (CES-D) [13-15,27,30]. The HADS is a self-administered questionnaire consisting of a 14 items, seven of which relate to anxiety and seven to depression [31]. A score of up to and including seven is classified as not depressed or anxious, those with a score from eight - 10 are defined as ‘possible’ depression or anxiety and a score of 11 or over was classified as ‘probable’ depression or anxiety. The CES-D [32,33] records the frequency and severity of depressive symptoms over the preceding week via a 20 item questionnaire. Each item is scored on a scale from 0 (less than 2 days duration) to 3 (5–7 days) inclusive, and a score of 16 or over indicates major depression.

Other tools used in the included papers were the Structured Clinical Interview (SCID-IV) for DSM-IV [26], the Geriatric Depression Scale (GDS) [25,29], the Goldberg Anxiety and Depression scale (GAD) [23], the International Classification of Diseases (ICD-9-CM) [24] and the Patient Health Questionnaire (PHQ) [22]. The SCID-IV is a standardised tool for diagnosing clinical depression and can diagnose both major and minor depression. The ICD-9-CM is more of a classification tool than a clinical instrument, but the study that reported use of this tool used it in conjunction with another tool, the Charlston Comorbidity Index (CCI). However it is not clear from the papers exactly how depression was measured using these instruments. The GDS [34] comprises a series of 30 yes/no questions about the patient’s feelings over the previous week. A score more than 15 (+/−6) indicates mild depression and a score over 23 (+/−5) major depression. The GAD [35] contains 18 questions, 9 each for anxiety and depression. Each of the items is scored by asking the patient to answer ‘yes’ or ‘no to a number of questions. Four questions for both anxiety and depression are used as ‘screening’ questions, and one or more positive responses to these questions leads to the interviewer asking the remaining questions. The higher a patient’s score, the higher the chance of them having clinically significant depression or anxiety. Finally, the PHQ [36] is a 10-item self-report questionnaire that asks patients to rate how often particular problems have bothered them over the preceding two weeks. At least four positive responses to any of the items are indicative of a depressive disorder, with a score of 20 or above indicating severe depression.

### Risk of bias within studies

Overall the papers scored highly on the CASP tools, with participant groups being recruited from acceptable populations. The studies used previously validated measures to collect data on anxiety and depression, although it was not always clear whether the measures had been validated within the study population. Not all studies specified cut-offs for major depression and none specified cut-offs for clinical anxiety, suggesting that precise prevalence estimates may not be available. The majority of included studies adjusted for potential confounding variables which could have an effect on the prevalence estimates obtained.

### Results of individual studies

All 16 included studies examined depression, with all of the articles consistently finding that the prevalence of depressive symptoms in adults with AMD was higher than in those without. The prevalence estimates provided ranged from 15.7%-44% in the cross-sectional and cohort studies and the case–control studies reported a significantly (*p* < 0.05) higher prevalence of depression in participants with AMD compared to non-AMD sufferers. Different methods were used to diagnose depression and studies included different subtypes of AMD, meaning a formal meta-analysis was not possible.

#### Depressive symptoms in neovascular AMD

Five publications studied depression in neovascular AMD specifically [3,19-21,28]. Of these, one study [28] was cross-sectional and the remaining four were case–control studies. All studies recruited their participants from eye clinics or specialist retina centres. All of the studies except one [28] compared their AMD sample depression prevalence rates with those of a community sample who did not suffer from any ocular pathology. The final study reported that they compared the findings from the AMD group with that of “a comparable German population” but did not give information as to the details of this population. The cross-sectional study reported a prevalence rate of 17.9% and the case–control studies all reported that depression was significantly (*p* < 0.05) more prevalent in the AMD populations. Three studies [19,20,28] looked at the relationship between AMD severity and depressive symptoms. Two [19,28] found that depressive symptoms worsen as neovascular AMD severity increases.

#### Depressive symptoms in non-specified AMD

The majority of studies did not specify whether they were examining neovascular or non-neovascular AMD. These studies recruited their participants mostly from eye clinics [13-15,23,25,27] or community settings [22,24,26,30], although one [29] did not state a setting. Six studies [13,14,23-26] stated that they compared their depression prevalence rates in AMD populations with community populations with no eye pathologies. One study [30] compared early and late AMD populations. For the remaining studies it was difficult to identify the sample they were comparing their findings in AMD populations against.

Cohort studies by Rovner et al. [13-15,22,27] reported prevalence estimates for depression of between 20% [22] and 43% [15]. Although the same data and method of measuring depression was utilised in all of the studies by this group, different figures of baseline depression prevalence were stated in the two articles that specified major depression (23.5% [13] and 27.5% [27]). The former study [13] found the prevalence to be much higher than normal for depression in older people attending primary care clinics, but included people with a much lower visual acuity (VA). One article [14] that used the same data as in a previous study [13] but a different method of calculating depression showed a higher prevalence (33%), suggesting the way in which depression is measured can affect study findings.

The case–control studies [23-25] that included a clinical cut-off found depression to be significantly (*p* < 0.05) higher in the AMD population than in the control group.

Two cross-sectional studies reported estimates of 26% [29] and 32.5% [26] although this latter estimate was lower when looking only at major depression (7.3%). A final cross-sectional study [30] reported prevalence estimates for depressive symptoms of 15.7% and 17.2% for early and late AMD respectively.

Few studies looked at whether severity of depressive symptoms was related to severity of AMD. Of those that did [14,27,29], all found that increasing AMD severity was related to an increase in depressive symptoms. A final study which compared the prevalence of depressive symptoms in those with early and late AMD [30] found no statistically significant difference between the two groups.

#### Anxiety symptoms in neovascular AMD

Four case–control studies examined anxiety in neovascular AMD, all of which recruited their participants from eye clinics. Three [3,20,21] reported no difference between those with AMD and those without. One [19] reported a prevalence which was significantly (*p* < 0.05) higher than the prevalence in a control population recruited from GP clinics. A cross-sectional study [28] found a prevalence estimate of 30.1% for anxiety. Of those that looked at the relationship between neovascular AMD severity and anxiety symptoms [19,20,28], none found a relationship.

#### Anxiety symptoms in non-specified AMD

One cross-sectional study [29] compared AMD patients with severe visual impairment with those with minimal impairment, and found no statistically significant difference (*p* = .13). Like the studies of neovascular AMD, this study also failed to find a relationship between an increase in anxiety symptoms and AMD severity.

### Risk of bias across studies

Only two papers were excluded for not being available in English (see Figure 1), suggesting that this is unlikely to have resulted in language bias. It is also possible that studies where a low prevalence of mental health symptoms was found were not published, leading to publication bias. However, a number of electronic databases were searched and reference lists were checked for published and unpublished literature in an attempt to locate all possible studies that had calculated prevalence estimates for AMD populations.

## Discussion

### Summary of evidence

The aim of this review was to assess the prevalence of symptoms of anxiety and depression in people with AMD. The results indicate that depressive symptoms are more common in people with AMD than in non-AMD populations. Overall, despite few studies looking into the association, there does appear to be a relationship between increasing AMD severity and higher prevalence of depression. Many of the studies did not differentiate between neovascular and non-neovascular AMD, and those that did found that both types of AMD were associated with depressive symptoms.

The results from the studies examining anxiety symptoms generally show that anxiety symptoms have a low prevalence in AMD populations. The two studies that did report a high prevalence [19,28] were large studies but one [19] did not report any cut-offs for their anxiety measure, so it was not clear if the anxiety measured was ‘clinically significant’. Overall, no study found a relationship between increasing AMD severity and anxiety prevalence in either the neovascular or non-specified AMD studies.

A number of issues with the included studies may help explain the range of different prevalence estimates found. The definition and measure of anxiety or depression used in each study and the type of AMD being investigated varied, making the results difficult to interpret collectively. Some studies compared their results to populations without AMD but did not actually include a control group within their study, which may mean that the comparison group is not representative. For example, the studies by Rovner et al. [13] and Casten et al. [15] provided prevalence estimates for depressive symptoms from older community populations (1-3% and 7.1% respectively), and Augustin et al. [28] stated that the prevalence of depression was higher than in a comparable German sample but did not provide the estimates for this sample or details of the population.

A final issue is that of causality, which cannot be established due to the design of the studies. Future observational studies could include an assessment of prior psychological distress, perhaps through a medical records review, in order to establish whether symptoms of anxiety or depression existed before the onset of AMD.

### Limitations

Many studies did not specify the type of AMD they were investigating, and from the small number of studies that looked specifically at neovascular AMD it is not possible to say whether the prevalence of symptoms of anxiety or depression are different in this particular form of the condition. The differentiation between the subtypes could be important due to the different time courses and treatments of each. The amount of time since diagnosis and how rapidly, and if, vision deteriorated was not mentioned in any of the studies. Ranibizumab is licensed for the treatment of neovascular AMD and a high success rate of maintaining patients’ current visual acuity has been reported [37]. The anticipation of vision deteriorating, how rapidly it has already deteriorated, the length of time since diagnosis and the possibility of available treatments are all important factors which could affect patients’ psychologically. The patient’s perception of how their vision loss impacts on their life is important to assess; a recent paper [38] found that self-reported vision loss was associated with depression where objective vision loss as assessed by an ophthalmologist was not. Further studies looking at AMD subtypes, the effect of length of time since diagnosis, and the treatment options offered or received to help improve patients’ mental health would be useful in helping decide when to consider psychological diagnoses and the most effective time to offer interventions.

While studies of RCTs would possibly have provided more clearly defined groups of AMD sufferers, they also contain more highly selected populations and tend to screen out co-morbidities such as symptoms of anxiety or depression, meaning that we may not have seen the true extent of these symptoms if we had included this type of study.

The CASP tool may not be appropriate to use with cross-sectional studies. However, the authors are not aware of any critical appraisal tool that is designed specifically for use with cross-sectional studies and it was decided that this tool would allow standardisation of the information collected and allow comparison across the studies. Also, the CASP tool provides an overall score for each study, which may not accurately compare study quality [5]. However, giving a summary score is helpful when gaining consensus between reviewers with respect to overall methodological quality and a detailed discussion of the critical appraisal can be found in the Results section.

Finally, many of the papers considered for inclusion in the review were located through additional searches and not through the electronic search strategy. However, the additional searches only located two more relevant papers, suggesting that the original search was adequate.

### Implications for practice

The evidence presented in this review supports evidence from previous studies that symptoms of depression are common in people with AMD. This indicates screening for depression should be considered, perhaps as part of routine follow-up, and appropriate resources allocated for this purpose. Further studies are needed to assess whether AMD sufferers would benefit from intervention options such as cognitive-behavioural therapy (CBT) or treatment with medication. The few studies that looked at whether severity of depressive symptoms was related to severity of AMD found that increasing AMD severity was related to an increase in depressive symptoms. Therefore interventions could be targeted at those with the most severe form of the condition. Again, more studies are required to differentiate between those with neovascular and non-neovascular subtypes as currently it is unknown whether depressive symptoms are more prevalent in one type of AMD. Since this review found little evidence for an increased risk of anxiety symptoms in AMD sufferers, anxiety screening is unlikely to be of benefit.

## Conclusions

The current literature examining the prevalence of symptoms of depression and anxiety in people with AMD suggests that depressive symptoms are prevalent in AMD populations compared with non-AMD populations, but anxiety symptoms are not. The studies included in the review scored well on the CASP tools, suggesting good methodological quality. However, many did not specify the type of AMD being assessed and different definitions of anxiety and depression were used, making the generalisability of the results difficult to assess. Future work would benefit from clearly specifying the type of AMD being studied, including a control or comparator group to allow for comparison of the prevalence of anxiety and/or depression between AMD and non-AMD populations, and the use of tools which give a clear cut-off for clinical anxiety and depression.

## Competing interests

The authors declare no financial or non-financial competing interests.

## Authors’ contributions

SRD, CDM, MBG and RY conceived the idea for the review. SRD, MBG and RY ran the search strategy and selected papers for inclusion. GM had full access to all the data in the study and takes responsibility for the integrity of the data and the accuracy of the data analysis. All authors extracted data and critically appraised included papers. SRD, CDM and GM drafted the manuscript. All authors read and approved the final manuscript.

## Pre-publication history

The pre-publication history for this paper can be accessed here:

http://www.biomedcentral.com/1471-2415/14/78/prepub

## Supplementary Material

Additional file 1: Table S1 Data extraction of all included studies. Click here for file
